# Prevalence of Renal Stones Among Patients With Inflammatory Bowel Disease in Saudi Arabia

**DOI:** 10.7759/cureus.15787

**Published:** 2021-06-21

**Authors:** Mahmoud Mosli, Abdulrahman M Alzahrani, Rafeef A Bahafzalla, Tala A Gazzaz, Rahaf M Slaghour, Ghidah Z Altabsh, Sarah B Aljadani, Razan N Alturkestani, Sondos S Hussein, Abdullah Kashgary, Omar I Saadah

**Affiliations:** 1 Department of Internal Medicine, Faculty of Medicine, King Abdulaziz University, Jeddah, SAU; 2 Faculty of Medicine, King Abdulaziz University, Jeddah, SAU; 3 Department of Pediatrics, Faculty of Medicine, King Abdulaziz University, Jeddah, SAU

**Keywords:** inflammatory bowel disease, renal stones, nephrolithiasis, prevalence, saudi arabia

## Abstract

Introduction: Crohn’s disease (CD) and ulcerative colitis (UC) are chronic inflammatory bowel diseases (IBD) that affect the gastrointestinal tract with no identified etiology. IBD has been associated with several extraintestinal manifestations (EIMs), including renal involvement such as renal stones (nephrolithiasis), resulting in significant morbidity. This study aims to estimate the prevalence of renal stones among IBD patients in Saudi Arabia.

Methods: This is a retrospective study conducted at King Abdulaziz University Hospital between January 2019 and December 2020. All IBD patients with abdominal imaging studies were included in the study regardless of their age. Data were collected from the electronic hospital information system and analyzed.

Results: A total of 363 IBD patients fulfilled the study inclusion criteria. Nephrolithiasis was detected radiologically in 3.6% of the cohort (5.1% of UC and 2.7% of CD patients). Patients with renal stones are older (P=0.002) and more likely to be diabetic (P=0.047), have microscopic hematuria (P<0.001), and proteinuria (P=0.002). Binary logistic regression analysis showed that older age at diagnosis (P=0.003) and microscopic hematuria (P=0.02) are independent predictors for renal stones.

Conclusion: The study reported that 3.6% of Saudi IBD patients had renal stones, with a higher prevalence of renal stones formation among UC patients than Crohn's. Older age at diagnosis and the presence of microscopic hematuria may predict the development of renal stones. Future studies should be conducted in a prospective manner at multiple centers across Saudi Arabia for further investigation.

## Introduction

Crohn’s disease (CD) and ulcerative colitis (UC) are chronic inflammatory bowel diseases (IBD) that affect the gastrointestinal tract with no identified etiology. The pathogeneses of IBD involve a dysregulated immune response to an environmental trigger in individuals with a genetic predisposition [1]. A systematic review that analyzed the global prevalence and incidence of IBD showed high prevalence rates in Europe and North America. Furthermore, the incidence in newly industrialized countries has been rising since 1990 [2]. Saudi Arabia showed a similar rise in IBD incidence, with a higher CD incidence than UC [3,4].

The clinical manifestations of IBD are consequences of ongoing inflammation, which is responsible for the progressive damage of the gastrointestinal tract. Symptoms suggestive of IBD include abdominal pain, diarrhea, rectal bleeding, anemia, fever, and weight loss [5]. IBD also has several extraintestinal manifestations (EIMs) involving the musculoskeletal system (arthritis and ankylosing spondylitis), skin (erythema nodosum, and pyoderma gangrenosum), and the eyes (Iritis and episcleritis). Among the EIMs of IBD, renal stones (nephrolithiasis) may develop in some IBD patients and result in significant morbidity [6]. The prevalence of nephrolithiasis varies between studies, with an estimated prevalence of 3% to 4.6% in European countries and up to 38% in Brazil and South American countries [7,8]. Renal involvement in IBD patients may lead to the development of renal insufficiency, which occurs in approximately 15.9% of patients [9].

We aim to estimate the prevalence of renal stones among IBD patients in Saudi Arabia, and to identify possible predictors of renal stone formation.

## Materials and methods

We conducted a hospital-based retrospective study of all patients with a confirmed diagnosis of IBD followed up at King Abdulaziz University Hospital (KAUH), Jeddah, Kingdom of Saudi Arabia, between January 2019 and December 2020. All IBD patients, regardless of their age, with reported abdominal ultrasonography (US), abdominal computed tomography (CT), or magnetic resonance imaging (MRI) were included in this study. All radiological reports are reviewed by a radiology consultant specialized in abdominal imaging. Patients' data, including clinical, demographic, and laboratory, were extracted from the medical records. The study was approved by the ethical committee at the Biomedical Ethics Unit at King Abdulaziz University (#581-19). The study was conducted in agreement with the Declaration of Helsinki.

Outcomes and definitions

The primary outcome of this study was to measure the prevalence of renal stones (defined as stones detected by US, CT, or MRI in the kidney, renal pelvis, or ureters) in IBD patients. The main secondary outcome of this study is to identify the clinical predictors and the risk factors contributing to the development of renal stones in IBD patients.

Statistical analysis

Baseline descriptive statistics were calculated for all patients. For continuous variables, means, standard deviations (SDs) were reported, and frequencies for categorical variables. Binary logistic regression analyses were used to identify predictors of primary and secondary outcomes. Odds ratios (OR) and 95% confidence intervals (CI) were estimated. Statistical Package for the Social Sciences version 26 (IBM Corp., Armonk, NY) was used in our analysis. A p-value of <0.05 is considered statistically significant.

## Results

Baseline characteristics

Totally, 363 patients (224 (6.32%) with CD and 137 (37.7%) with UC) who fulfilled the inclusion criteria were included in this study. The median age was 32 years (range, 6-84 years). Females constituted 52.9% (n=192), and 84.6% were older than 18 years of age. Associated comorbidities comprising hypertension in 5.5% and diabetes mellitus in 6.6%. Details of baseline characteristics are shown in Table 1.

**Table 1:** Baseline characteristic of the study cohort *Chi-square or student t-test. **P<0.05.

Variable	Total N=363	UC N=137	CD N=226	P-value*
Age at diagnosis (years), mean±SD	34.78±15.9	40.1±18	31.58±13.6	<0.001**
Age category				
0-18 years	56 (15.4%)	22 (16.1%)	34 (15%)	0.88
>18 years	307 (84.6%)	115 (83.9%)	192 (62.3%)	
Gender				
Male	171 (47.1%)	59 (43.1%)	112 (49.6%)	0.24
Female	192 (52.9%)	78 (56.9%)	114 (50.4%)	
Nationality				
Saudi	246 (67.8%)	77 (56.2%)	169 (74.8%)	<0.001**
Non-Saudi	117 (32.2%)	60 (43.8%)	57 (25.2%)	
Hypertension				
Yes	20 (5.5%)	6 (4.4%)	14 (6.2%)	0.64
No	343 (94.5%)	131 (95.6%)	212 (93.8%)	
Diabetes mellitus				
Yes	24 (6.6%)	8 (5.8%)	16 (7.1%)	0.83
No	339 (93.4%)	129 (94.2%)	210 (92.9%)	
Hemoglobin (g/dL)	11.7±2.3	11.7±2.5	11.8±2.2	0.85
C-reactive protein (mg/dL)	22.9±43.2	25.4±48.9	21.3±39.6	0.41
Albumin (g/L)	31.4±7.9	33.2±7.3	30.3±8	0.001**

Study outcomes

Of the total cohort, 13 (3.6%) had renal stones; 5.1% of UC patients (n=7) and 2.7% of CD patients (n=6). Stones were detected by the US in nine patients and by CT scan in four patients. It was single in 12 patients and multiple in two other patients. The characteristics of renal stones are shown in Table 2.

**Table 2:** The characteristics of the inflammatory bowel disease patients with renal stones CD: Crohn’s disease, UC: ulcerative colitis, US: ultrasonography.

Number	Gender	IBD subtype	Imaging detection	Stone numbers	Stone size (mm)	Stone location	Associated hydronephrosis or hydroureter	Associated anomalies
1	M	UC	CT	Single	2	Right lower pole	No	Bilateral renal cysts
2	M	UC	US	Single	7	Left lower pole	No	No
3	F	UC	US	Single	4	Left lower pole	No	Right cortical cyst; left cortical calcification
4	M	CD	US	Single	8	right	No	No
5	F	CD	CT	Single	4	Left ureter	Mild hydroureter and hydronephrosis	No
				Multiple	Tiny	Renal bilateral		
6	F	CD	US	Single	8	Right lower pole	No	No
7	F	UC	CT	Multiple	Tiny	Bilateral renal	No	Bilateral simple renal cysts
8	M	UC	CT	Single	8	Left lower pole	No	Right renal cyst
9	F	CD	US	Single	3.6	Right renal	No	No
10	F	UC	US	Single	3.2	Left mid pole	No	No
11	F	CD	US	Single	6	Left upper pole	Slight calyceal fullness	No
12	M	UC	US	Single	4	Right lower pole	No	No
13	F	CD	US	Single	8	Rt upper pole	No	No

Comparing patients with and without renal stones (Table 3) revealed that patients with renal stones are older (P=0.002), had more DM (P=0.047), and more likely to have microscopic hematuria (P=0.002) and proteinuria (0.002) in urinary examinations.

**Table 3:** Comparing patients with and without renal stones *Chi-square, Fisher’s exact, or student t-test. **P<0.05.

Variables	Renal stone N=13 N (%) or mean±SD	No renal stone N=350 N (%) or mean±SD	P-value*
Age at diagnosis (years)	48.5±16.7	34.3±15.7	0.002
Gender			
Male	5 (38.5%)	166 (47.4%)	0.58
Female	8 (61.5%)	184 (52.6%)	
IBD subtypes			
CD	6 (46.2%)	220 (62.9%)	0.25
UC	7 (53.8%)	130 (37.1%)	
Nationality			
Saudi	8 (61.5%)	238 (68%)	0.76
Non-Saudi	5 (38.5%)		
Chronic kidney disease			
Yes	0 (0.0%)	3 (0.9%)	1.0
No	13 (100%)	347 (99.1%)	
Diabetes mellitus			
Yes	3 (23.1%)	21 (6%)	0.047**
No	10 (76.9%)	329 (94%)	
Hypertension			
Yes	18 (5.1%)	2 (15.4%)	0.16
No	332 (94.9%)	11 (84.6%)	
Renal malignancy			
Yes	1 (7.7%)	1 (0.3%)	0.07
No	12 (92.3%)	349 (99.7%)	
Microscopic hematuria			
Positive	9 (69.2%)	56 (16%)	<0.001**
Negative	4 (30.8%)	294 (84%)	
Proteinuria			
Positive	6 (50%)	42 (12%)	0.002**
Negative	6 (50%)	308 (88%)	
Fistula			
Yes	1 (7.7%)	36 (10.3%)	1.0
No	12 (92.3%)	314 (89.7%)	
CRP (mg/dL)	10.8±10.2	23.2±43.9	0.37
ESR (mm/H)	35.4±34.5	28.5±25.2	0.48
Creatinine (µmol/L)	72.6±27.8	64.5±27.7	0.31
Hb (g/dL)	12.5±2.4	11.7±2.3	0.25
Albumin (g/L)	33.2±7.5	31.4±7.8	0.41

Binary logistic regression analysis identified older age at diagnosis (P=0.003) and the presence of microscopic hematuria (P=0.016) as predictors for renal stones (Table 4).

**Table 4:** Binary logistic regression analysis of possible predictors of renal stones *P<0.05. OR: odds ratio.

Variable	B	SE	OR	95% CI for OR		P-value
				Lower	Upper	
Age at diagnosis (years)	0.058	0.019	1.06	1.02	1.10	0.003*
Diabetes mellitus	0.743	0.91	2.10	0.35	12.53	0.41
Microscopic hematuria	1.836	0.77	6.27	1.39	28.12	0.02*
Proteinuria	1.330	0.78	3.78	0.81	17.55	0.09

## Discussion

This study estimated the prevalence of renal stones among IBD patients in a single tertiary care center in Jeddah, Saudi Arabia. Renal involvement is considered one of the rare EIMs of IBD, which occurs in 4-23% of patients [10]. In addition, renal involvement in IBD may negatively impact the patient's quality of life [11]. The current study estimated that 3.6% of IBD patients have renal stones, which is lower than the prevalence rate reported in the United States of 12-28% [12]. This variation could be attributed to differences in the disease severity of the population studied and the methods of detection. In severe disease, the amount of extracellular fluid loss due to diarrhea and malabsorption may contribute to the development of renal stones [13,14]. Our results showed a higher prevalence of renal stone formation among UC patients compared to CD (5.1% vs. 2.7%). This result is different from the findings of previously reported studies of a higher prevalence of renal stones in CD patients than in UC [7,14-17]. Studies reported increased risk in UC patients after Ileoanal pouch anastomosis [18,19]. This may be attributed to the crystallized precipitation of mesalazine - a commonly used medication for UC - as reported in the literature [20-24].

In the present study, we found no gender influence on renal stone formation among IBD patients. This finding is in accordance with the results of another study from the United Kingdom [14]. Other studies have reported male predominance in both UC and CD [7], and in CD only [25]. Older age at diagnosis, DM, and microscopic hematuria were identified as possible predictors for renal stones formation. Previous studies have reported the association of DM with nephrolithiasis [26-28]. Therefore, the coexistence of DM and IBD may increase the risk of developing renal stones. We also found microscopic hematuria among the predictors for the development of renal stones. This may result from local tissue damage owing to the presence of renal stones [29] or may be considered as an EIMs of IBD per se, as previously reported [30]. We did not find proteinuria as a predictor for renal stone formation. It rather reflects other renal pathology such as tubulointerstitial nephritis [31-38].

The study may be limited by its retrospective nature, lack of control group, and a lack of long-term follow-up, but sheds significant light on the occurrence of renal stones in Saudi patients with IBD patients that may add more to their existing morbidity.

## Conclusions

Nephrolithiasis is known as one of the EIM of IBD. The study showed that 3.6% of Saudi IBD patients had nephrolithiasis with high prevalence of renal stone formation among UC patients compared to CD. Older age at diagnosis and microscopic hematuria were identified as predictors for developing kidney stones. While other factors were not found to be predictors, future studies should be conducted in a prospective manner at multiple centers across Saudi Arabia for further investigation.
